# Positive attitudes toward adoption of a multi-component intervention strategy aimed at improving HIV outcomes among adolescents and young people in Nampula, Mozambique: perspectives of HIV care providers

**DOI:** 10.1186/s12913-023-09630-1

**Published:** 2023-06-06

**Authors:** Phepo Mogoba, Maia Lesosky, Elton E. Mukonda, Allison Zerbe, Joana Falcao, Ricardino Zandamela, Landon Myer, Elaine J. Abrams

**Affiliations:** 1 Division of Epidemiology & Biostatistics, School of Public Health & Family Medicine, University of Cape Town, Level 5, Falmouth Building, Anzio Road, Cape Town, South Africa; 2 ICAP at Columbia University, Mailman School of Public Health, New York, USA; 3 ICAP at Columbia University, Maputo, Mozambique; 4 Department of Pediatrics, Vagelos College of Physicians and Surgeons, Columbia University, New York, NY USA

**Keywords:** Adolescents, Young adults, HIV, Multi-component intervention, Adoption, Attitudes, Implementation, Sub-Saharan Africa

## Abstract

**Background:**

Service providers' attitudes toward interventions are essential for adopting and implementing novel interventions into healthcare settings, but evidence of evaluations in the HIV context is still limited. This study is part of the CombinADO cluster randomized trial (ClinicalTrials.gov NCT04930367), which is investigating the effectiveness of a multi-component intervention package (CombinADO strategy) aimed at improving HIV outcomes among adolescents and young adults living with HIV (AYAHIV) in Mozambique. In this paper we present findings on key stakeholder attitudes toward adopting study interventions into local health services.

**Methods:**

Between September and December 2021, we conducted a cross-sectional survey with a purposive sample of 59 key stakeholders providing and overseeing HIV care among AYAHIV in 12 health facilities participating in the CombinADO trial, who completed a 9-item scale on attitudes towards adopting the trial intervention packages in health facilities. Data were collected in the pre-implementation phase of the study and included individual stakeholder and facility-level characteristics. We used generalized linear regression to examine the associations of stakeholder attitude scores with stakeholder and facility-level characteristics.

**Results:**

Overall, service-providing stakeholders within this setting reported positive attitudes regarding adopting intervention packages across study clinic sites; the overall mean total attitude score was 35.0 ([SD] = 2.59, Range = [30–41]). The study package assessed (control or intervention condition) and the number of healthcare workers delivering ART care in participating clinics were the only significant explanatory variables to predict higher attitude scores among stakeholders (β = 1.57, 95% CI = 0.34–2.80, *p* = 0.01 and β = 1.57, 95% CI = 0.06–3.08, *p* = 0.04 respectively).

**Conclusions:**

This study found positive attitudes toward adopting the multi-component CombinADO study interventions among HIV care providers for AYAHIV in Nampula, Mozambique. Our findings suggest that adequate training and human resource availability may be important in promoting the adoption of novel multi-component interventions in healthcare services by influencing healthcare provider attitudes.

## Background

In recent years, public health researchers across different domains have recognized the importance of planning and developing health interventions responsive to the healthcare needs of priority populations to ensure their impact where they are delivered [1–4]. However, the success of needs-based interventions is not solely determined by how they are developed; it also depends on how well they are implemented. Successful implementation of novel evidence-based interventions (EBIs) is vital for ensuring that such interventions can reproduce lasting, effective health outcomes in the intended settings [5]. With the evolution of implementation science, researchers can now develop strategies that support the effective and timely translation of EBIs into practice [6, 7]. The advances are crucial, particularly in sub-Saharan Africa (SSA), where many efforts are being made to develop effective and contextually appropriate interventions to improve HIV-associated health outcomes [8–15]. Moreover, policymakers find evidence from such work relevant in determining which interventions to scale up, especially in resource-constrained situations.

According to implementation literature, implementing new interventions into practice is complicated since it involves a series of interlinked phases, such as pre-implementation (adoption), implementation, and post-implementation (sustainability) [16]. As part of the implementation process continuum, interventions introduced into a new setting must be adopted effectively by individual providers and organizations that will deliver them. Proctor and colleagues define adoption as the “intention, initial decision, or action to try or employ an innovation or evidence-based practice” [17]. Based on this definition, any challenges with provider or organization decisions to adopt an intervention in the early stages of implementation may negatively affect the later stages of the implementation process and, consequently, the overall success of an intervention [18]. Given this viewpoint, researchers should identify early in the implementation process the factors that may affect the adoption of new interventions in order to develop effective context-specific strategies to increase their adoption. Although, in recent years, there has been an increase in empirical studies that have focused on intervention adoption across different research disciplines, data within the HIV context remains limited [19–30]. Moreover, since most of these studies were conducted in high-income countries, owing to differences in contexts, their findings do not apply to SSA, where evidence from such studies may be particularly beneficial.

A review on adoption by Wisdom et al. has highlighted the importance of attitudes of individual providers toward an intervention in influencing intervention adoption [18]. Positive provider attitudes appear to be associated with an increased likelihood of adoption and subsequent use of new interventions [18, 31, 32]. The premise is that even if an organization adopts a novel EBI in practice, if there is a lack of acceptance among service providers, EBI may either not be used or may not be used as intended [19]. Furthermore, studies indicate that providers' attitudes toward implementing interventions are influenced by several complex factors related to the intervention (e.g., content and structure), the context in which the intervention is delivered (i.e., setting, organization, political climate, etc.), and provider characteristics [19, 33–35]. Therefore, understanding the role of these factors may be crucial for any new intervention implementation effort.

The CombinADO study (ClinicalTrials.gov NCT04930367) is a cluster-randomized trial (cRCT) of a novel multi-component HIV care package that aims to improve HIV outcomes such as viral suppression, retention, and adherence to ART among adolescents and young adults living with HIV (AYAHIV), ages 10–24 years, in routine HIV care in Nampula, northern Mozambique. Following the successful participatory development of the study interventions based on the identified needs surrounding the treatment of AYAHIV within this setting, we sought to understand the attitudes of stakeholders providing HIV care among AYAHIV in adopting interventions within health facilities [36–38]. In addition, we aimed to identify factors that would need attention to support effective implementation. In this paper, we present findings on attitudes of key stakeholders towards adopting CombinADO intervention packages in HIV care facilities for AYAHIVs. In addition, we report on the impact of stakeholder and facility-level characteristics on stakeholders' attitudes.

## Methods

### CombinADO study

A detailed description of study interventions and methods of the CombinADO trial has previously been published elsewhere [37]. Briefly, the CombinADO intervention strategies have been developed as a response to the National Institute of Child Health and Development (NICHD) call for more research towards generating needed evidence on effective public health interventions for young people affected by HIV in resource-limited settings, the Prevention and Treatment through a Comprehensive Care Continuum for HIV-affected Adolescents in Resource-Constrained Settings (PATC^3^H) program [39]. This study is a result of the collaborative efforts between the PATC^3^H consortium, the Mozambique Ministry of Health (MISAU) and researchers from ICAP at Columbia University to evaluate a complex, multi-component intervention to improve HIV-related health outcomes of AYAHIV in Mozambique (CombinADO strategies). The trial is conducted in Nampula Province, a low-resource setting in northern Mozambique. The HIV prevalence among young people 15–24 years is estimated at 4.1% in Nampula, with only 16% of clinics in this province currently offering the national package for adolescent and youth-friendly services (AYFS). The national AYFS includes HIV prevention, care, and treatment; screening and treatment for sexually transmitted infections (STIs); pre-natal and post-natal care; family; intimate partner violence (IPV) services and information on other diseases [40]. In this trial, 12 health facilities across Nampula Province providing HIV care to AYAHIV were randomized either to deliver the CombinADO intervention package (herein, intervention) or the enhanced standard of care (ESOC) intervention package (herein, control). Each study condition includes delivery of a multi-component package of intervention modalities within participating HIV care sites for AYAHIV. The description and rationale of the set of intervention components included in each study condition is outlined in Table 1. Approximately 10,370 AYAHIV were receiving routine ART care at various HIV care service sites across the 12 participating health facilities, including in AYFS, where the CombinADO strategies were implemented. As part of the process of generating evidence of the effectiveness of the intervention packages, the study is collecting data on the implementation process to facilitate efficient and successful implementation of strategies in facilities in the future if they are found successful.

**Table 1:** CombinADO package components during the 12 months of implementation by study arm

Component	Rationale	Study arm	
		**Control**	**Intervention**
**Radio ads**	Engaging radio mini shows that address community stigma and medical literacy through busting common myths with humour and building empathy with heartfelt storytelling	X	X
**Community sensitization campaign**	Large-scale, infographic billboards and posters located in public areas and secondary schools to address stigma, medical literacy and promote community support for AYAHIV	X	X
**Informational posters**	Large-scale, infographic posters located in clinic waiting areas to normalize HIV and build confidence in treatment	X	X
**Motivation walls**	Interactive, patient-generated posters located in the consultation room where patients can post words and phrases about themselves and their futures	X	X
**Pill boxes**	A discreet pill container to support ART adherence	X	X
**CombinADO-specific AYAHIV training**	Comprehensive in-service training for healthcare workers	X	X
**One-stop shop**	Combined adolescent and HIV services	X	X
**Treatment toolkit**	A guide to clinic visits and discussions on Art and viral load monitoring to help HCW better communicate with patients	X	X
**Self-Reflection kit**	A simple handout for providers to help patients reflect on their ART progress and understand the concept of viral load as a measure of ART success	X	X
**Peer support at clinical level**	Peer exposure to examples of AYAHIV openly living with HIV and opportunities to share their experiences with HIV in one-on-one interactions with other AYAHIV during clinic visits		X
**Informational and motivational video**	An informational and motivational video that in simple language with engaging graphics that a) demystifies and simplifies HIV, ART, and viral load and b) emphasizes that people can live long, healthy lives		X
**Support groups for caregivers of AYAHIV**	A learning, support, and empowerment group for caregivers of AYAHIV. Through monthly gatherings, the program aims to foster confidence, and equip caregivers with strategies to support AYHIV adherence journey		X
**Support groups for AYAHIV**	A peer-to-peer learning, support, and empowerment group to address loss of hope and improve medical literacy. Through biweekly gatherings, the program aims to foster belonging and confidence, equipping young people and caregivers with strategies to navigate the adherence journey		X
**Mental health screening and linkage to adolescent-focused mental health support**	HCWs will be trained in the use of a brief mental health screening tool focusing on depression, anxiety, and post-traumatic stress disorder. Mental health service providers at each facility will be trained and supported to provide diagnostic and mental health support to youth with positive screens who agree to further evaluation		X

### Study design

This cross-sectional study employed data drawn from surveys conducted in the pre-implementation phase of the CombinADO trial between September and December 2021. The study was designed to collect both quantitative data (surveys) and qualitative data (IDIs). Prior to the pre-implementation survey, participants were invited to a two-day workshop with the objective of sensitizing stakeholders at the various research sites to the study's goals and introducing them to the study materials. As part of the awareness-raising efforts, the study team 1) conducted information session on health services needs for AYHIV 2) presented the CombinADO study, 3) shared information about Phase 1 (i.e., participatory development phase of intervention packages), and 4) provided plans for Phase 2 implementation in the 12 study sites to both stakeholders representing both control and intervention sites as identified by study site leaders on day 1 of the workshop. During the same session, the study team introduced the study materials that were planned for implementation uniformly in both the control and intervention sites. This was supplemented with role play for the materials, and the session lasted the entire day (~ 8 h). Only stakeholders from the intervention sites were invited to another session (~ 4 h) on day 2 of the program for further introduction of the other six materials to complete presentations of the intervention package. This session did not cover any more information other than the introduction of material, to ensure that stakeholders from both the control and intervention sites got equal and relevant information about the study prior to evaluating stakeholder views. On both days of the workshop, the sessions were led by the same trainer.

### Participants

Participants included a purposive sample of 59 stakeholders involved in implementing [ i.e., health care workers (HCWs), *n* = 26] and overseeing [i.e., key informants (KIs), *n* = 33] HIV services for AYAHIV in the 12 health facilities participating in the CombinADO study. Only two (the largest in the city) of the 12 participating facilities had previously taken part in piloting the study packages during the participatory development of study packages in Phase 1. To be able to include the relevant stakeholders for this study, we leaned on expertise of directors from study sites to identify stakeholders within each facility who would represent the diverse occupational structures of stakeholders providing HIV services to AYAHIV. When stakeholders were confirmed by study team to meet the criteria, they were then invited to a two-day workshop. Among the stakeholders who attended this workshop were HCWs ―nurses, doctors― and KIs made up of ICAP project staff (*n* = 13), health facility directors (*n* = 16), and staff from regional health offices (*n* = 3) and MISAU (*n* = 1) all of whom were having some level of clinical training. Eligible study participants were identified and were recruited face-to-face by a trained interviewer during the two-day workshop with the health facilities and the MOH that aimed to introduce the study and study material to stakeholders. Eligibility criteria included (1) being 18 years and older, (2) involvement in the provision, management, or oversight of adolescent-focused HIV services at the 12 specified study sites, and (3) willingness to be audio-recorded in IDIs. All interested stakeholders were invited for a face-to-face interview that took part within a few days of the two-day workshop, depending on their availability and capacity of the interviewer. Interviews were conducted in a private place, and all participants provided written informed consent. All surveys and IDIs were conducted in Portuguese by one trained interviewer, and all IDIs were audio-recorded. All stakeholders had no prior knowledge of the study packages, apart from five stakeholders (2 from a control site; 3 from an intervention site) who worked at the two facilities that piloted the study packages in Phase 1.

### Measurements

In this study, the RE-AIM framework guided the evaluation of the pre-implementation phase of the CombinADO strategies [41]. The survey measures were formulated from quantitative items adapted from existing, validated measures recommended by the PATC^3^H IS working group for assessing three RE-AIM constructs: adoption, implementation, and maintenance [24, 42–44]. Survey data were collected to understand stakeholder attitudes toward adoption, initial willingness to implement, and perspectives on maintenance of the study strategies before intervention packages were implemented at sites. Since both HCWs and KIs were eligible to participate in the interviews, the study team tailored the survey questions to reflect their different roles within their clinics and the different study conditions in the clinics. Given that these original measures were available only in English, they were translated into Portuguese and back-translated into English by English-Portuguese research team members. This paper presents the findings from analyses of the survey data related to the adoption construct.

#### Attitudes towards intervention package adoption

Stakeholder attitudes toward the adoption of the CombinADO strategies were assessed using an Attitudes towards intervention subscale previously developed in the Antiretroviral Treatment Access Study (ARTAS), which aimed to evaluate the adoption of an evidence-based HIV linkage-to-care intervention among AIDS directors in the United States [24, 44]. In this study, the attitude towards the adoption endpoint is derived as a continuous composite score based on 9 of the 17 original subscale items assessed among stakeholders in implementing sites. Responses for each of the items were scored on a five-point Likert scale of 1 (strongly disagree) to 5 (strongly agree). The scores of each item were summed, and total scores can range from a minimum of 9 to a maximum of 45 with a midpoint of 27. Two items within this scale are negatively framed and thus were reverse scored before computing the total score. The total score represents one's attitude toward the adoption of the intervention package. Following the approach used by Norton, higher total scores indicated a more positive attitude towards the adoption of intervention packages in health facilities, as reported by stakeholders [24]. For stakeholders scoring below the 27 these could be said to have overall less positive attitudes, whereas those scoring above 27 considered as generally showing positive attitudes towards the adoption of the intervention package.

#### Explanatory variables

Stakeholder-level characteristics were collected on a brief questionnaire which included self-reported gender, age, professional title, primary role in the clinic (HCW vs KI), and years worked at the facility (HCWs only). Facility-level characteristics associated with stakeholders were collected, and these included the location of the facility (i.e., urban vs rural vs peri-urban vs governmental workplace), Number of HCWs in AYAHIV services (i.e., number of HCWs delivering ART services to AYAHIV at clinics), and patient volume (i.e., number of AYAHIV receiving HIV care at the facility). Our consideration of these variables as potential predictors of stakeholders' attitudes toward adopting intervention packages was based on their consideration in several studies that examined attitudes toward EBIs among stakeholders in different health research topics and warranted examination in this context [24, 45].

### Data analysis

This analysis was conducted using Stata version 14.2 (Stata Corp, College Station, TX, 176 USA). Stakeholder and facility-level characteristics were summarised using frequencies, proportions, means and standard deviations (SD) or medians and their interquartile ranges (IQR), stratified by intervention group (i.e., CombinADO package and ESOC package). Bivariate analyses for comparison of predictor variables and attitude scores across intervention groups were performed using a t-test or Wilcoxon rank-sum test for continuous data and Chi-square or Fisher's exact test as appropriate for categorical data.

Regression analyses were considered to understand the association of total attitude scores with stakeholder and facility-level characteristics. Given that the data from this cross-sectional study were embedded within a cRCT, we first had to consider the effect of clustering of the stakeholders―stakeholders being nested within clinics―on reported attitude scores to ensure results were not biased. We estimated a null model (i.e., containing no explanatory variables) to test for random effects of within and between clinic variations by computing an intraclass correlation (ICC). The model revealed that respondents' attitude scores were not influenced by site clustering, thus showing that multilevel modelling may not be warranted. We used simple linear regression models to identify explanatory variables that significantly predicted stakeholders' attitude scores for adopting the intervention packages. Finally, we fitted a multivariable linear regression model that included explanatory variables found to be significant at *p*-value < 0.10 in univariate models. The results were presented as β-coefficients of unadjusted and adjusted linear regression and their 95% confidence intervals (CI). A *p*-value < 0.05 was considered statistically significant for all tests except for simple linear regressions.

### Ethics

The study was approved by the Columbia University of Irving Medical Center Institutional Review Board (CUIMC IRB# AAAT5971) and the Comite Nacional De Bioética Para a Saúde de Moçambique (467/CNBS/21).

## Results

### Stakeholder and facility-level characteristics

Summaries of individual-level and facility-level characteristics of stakeholders are presented in Table 2. Among the 59 stakeholders who participated in the survey, the majority were female (*n* = 41, 69%), with a mean age of 34.7 (SD = 0.88) years, ranging from 22 to 50 years. Regarding the primary role in clinics, 33 (56%) were KIs, and 26 (44%) were health care workers (HCWS) at clinics, with HCWs working in clinic facilities for a median of 2 years (IQR: 1–8). The study sample included nurses/midwives (*n* = 21, 36%), medical doctors (*n* = 18, 30%), and health techs (*n* = 17, 29%), with only a few working in other professions (*n* = 3, 5%). Regarding the geographic distribution of clinics represented by stakeholders, the majority (*n =* 29, 49%) were in urban areas, and only a few (*n* = 4, 7%) stakeholders represented governmental workplaces not located directly in the facilities. On average, these AYFS clinics served a median of 198 AYAHIV (IQR: 152–287) in facilities, with a median of 2 HCWs (IQR: 2–3) providing ART care at these AYFS at a range of 1 to 3 HCWs per facility.

**Table 2:** Individual and facility-level demographic characteristics for stakeholders by intervention package grouping, (*N* = 59)

Characteristic	Total (*N* = 59)	CombinADO package (*N* = 30)	ESOC package (*N* = 29)	*p*-value
***Individual level***				
**Age, years, mean (SD); range**	34.7 (0.88); 22–50	35.1 (7.04); 24–48	34.2 (6.61); 22–50	0.60
**Gender**				0.22
Male	18 (31)	7 (23)	11 (38)	
Female	41 (69)	23 (77)	18 (62)	
**Primary role at clinic**				0.52
Health care worker (HCW)	26 (44)	12 (40)	14 (48)	
Key informants (KI)	33 (56)	18 (60)	15 (52)	
**Current position**				0.39
Medical doctor	18 (30)	9 (30)	9 (31)	
Nurse/midwife	21 (36)	11 (37)	10 (34.5)	
Health tech	17 (29)	7 (23)	10 (34.5)	
Other profession	3 (5)	3 (10)	0 (0)	
**Years working in the facility, median (IQR); range, *n* = 26**^**a**^	2 [1,8]; < 1–13	1 [1,3]; < 1–6	6 [1,10]; < 1–13	**0.02**
***Facility level***				
**Location of facility**				** < 0.001**
Rural	18 (31)	9 (30)	9 (31)	
Urban	29 (49)	9 (30)	20 (69)	
Peri-urban	8 (13)	8 (27)	0 (0)	
Governmental KI	4 (7)	4 (13)	0 (0)	
**Facility size, median (IQR); range, *n* = 55**^**b**^	198 [152, 287]; 100–643	176 [148, 287]; 125–643	215 [162, 221]; 100–334	0.58
**HCWs delivering ART services to AYAHIV in the facility, median (IQR); range**	2 [2, 3]; 1–3	2 [2, 3]; 1–3	2 [2]; 1–3	0.44
1 HCW	8 (14)	4 (15)	4 (14)	
2 or more HCWs	47 (86)	22 (85)	25 (86)	

*SD* Standard deviation, *IQR* Interquartile ranges, Results are n (column %) with *p*-value from chi^2^ test and Fisher’s exact test, Mean (SD) and median (interquartile range, IQR) with *p*-value from t-test and Wilcoxon rank-sum test

The bold text under the *p*-value column shows statistical significance

^a^Data only collected in HCWs

^b^Data only for facility-based stakeholders

### Demographic comparisons by intervention status

Of the 59 stakeholders surveyed, 30 (51%) represented CombinADO package clinics and 29 (49%) on the ESOC package clinics, with only a few significant differences in characteristics identified between these groups (Table 2). The HCWs from clinics surveyed on the ESOC package reported significantly longer years of working in clinics compared to those surveyed on the CombinADO package [(Median (IQR): 6 [1–10] vs 1 [1–3], respectively; *p* = 0.02)]. A significantly higher number of stakeholders in the ESOC package group (*n* = 20, 69%) represented urban-based clinics compared to a lower number (*n* = 9, 30%) of those in the CombinADO package group who were from a similar setting, *p* < 0.001. The rest of the CombinADO package stakeholders represented clinics located in the rural (*n* = 9, 30%) and peri-urban (*n* = 8, 27%), governmental key informants (*n *= 4, 13%), while the remaining (*n* = 9, 31%) ESOC package stakeholders represented rurally based clinics.

### Patterns of stakeholder attitude scores

**Fig. 1:**
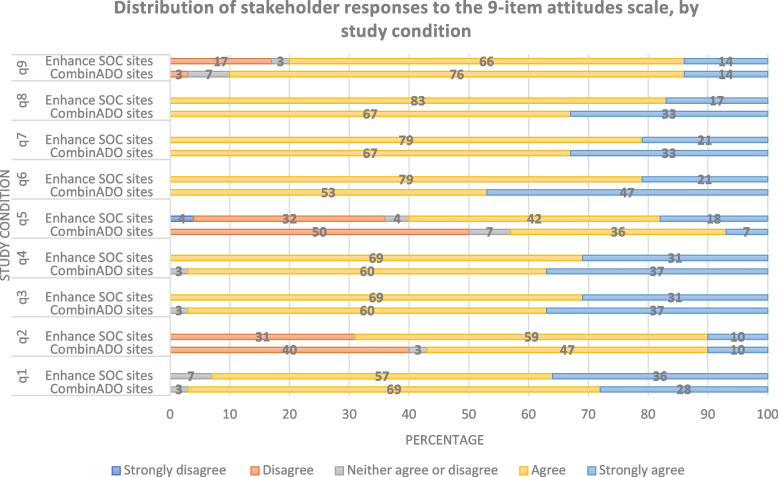
A total of 100% stacked bar charts showing the distribution of the stakeholder Likert scale responses to the 9 items assessing attitudes towards the adoption of study intervention packages in clinics by study condition [i.e., Enhanced SOC (*n* = 29); CombinADO(*n* = 30)]. Refer to Q1–Q9 of Table 3 for the wording of the questions represented in this figure

The overall mean (M) total attitude score was 35.0 ([SD] = 2.59, Range = [30-41]), indicating that most stakeholders had a relatively positive attitude toward the adoption of intervention packages across sites. On average, stakeholders in CombinADO package clinics reported more positive attitudes towards the intervention package (M = 35.8, [SD] = 2.31) than those in enhanced SOC clinics (M = 34.2, [SD] = 2.65), *p* = 0.02. There were no significant differences in distributions of stakeholders' rating of item responses by intervention group, except for Q6 of the measure (Table 3 & Fig. 1). A significantly higher number of stakeholders in the CombinADO intervention package, 47%, stated that they strongly agreed that the designated intervention package would be easy to understand and use after training, compared to only 21% who felt the same way about the ESOC intervention package (*p* = 0.04).

**Table 3:** Mean item and total stakeholder attitudes scores towards adoption of intervention packages, overall and by study condition, (*N* = 59)

Items		Mean score (SD)			*p*-value
		**Total (*n* = 59)**	**Intervention sites (*n* = 30)**	**Enhance SOC sites (*n* = 29)**	
**Q1**	**The* intervention* would be more effective than interventions currently being used to improve retention and viral suppression among AYLHIV**	4.3 (0.55)	4.3(0.60)	4.24 (0.51)	0.69
**Q2**	**The *intervention package* is too complex**^**a**^	2.62 (1.08)	2.7 (1.11)	2.5 (1.05)	0.45
**Q3**	**The *intervention package* would be successful to improve retention and viral suppression among AYLHIV this clinic**	4.37 (0.52)	4.4 (0.57)	4.3 (0.47)	0.37
**Q4**	**The *intervention package* is compatible and consistent with the needs of AYLHIV**	4.32 (0.51)	4.3 (0.55)	4.3 (0.47)	0.86
**Q5**	**The *intervention package* requires too many human resources, *n* = 58**^**a**^	2.8 (1.16)	3.0 (1.08)	2.6 (1.23)	0.20
**Q6**	**The *intervention package* would be easy to understand and use after training**	4.3 (0.48)	4.5 (0.51)	4.2 (0.41)	**0.04**
**Q7**	**The *intervention package* would have a visible and substantial impact on the health status of AYLHIV in this clinic**	4.3 (0.45)	4.3 (0.48)	4.2 (0.41)	0.28
**Q8**	**AYLHIV would benefit from the *intervention package***	4.3 (0.44)	4.3 (0.48)	4.2 (0.38)	0.16
**Q9**	**The *intervention package* could be easily adapted to fit the needs of community-based organizations and/or health departments that would implement it, *n* = 58**	3.9 (0.77)	4.0 (0.60)	3.8 (0.91)	0.24
**Mean Total Attitude score (SD); range**^**b**^		35.0 (2.59); 30–41	35.8 (2.31); 31–41	34.2 (2.65); 31–41	**0.02**

Note. 1 = Strongly Disagree, 2 = Disagree, 3 = Neither Agree nor Disagree, 4 = Agree, 5 = Strongly Agree

The bold text under the *p*-value column shows statistical significance

^a^ Items were reverse scored before conducting test statistics and creation of the average score

^b^ Higher score indicates more positive attitudes towards intervention package

### Associations of individual stakeholder and facility-level characteristics with attitude score

Table 4 presents the results of models from univariate and multivariable-adjusted models. The intraclass correlation coefficient for attitude scores was ICC = 0.04, with an estimated 4.4% total variation attributable to the facility. On the basis of this, we elected to use generalized linear regression. Univariate analyses showed that significantly more positive total attitude scores were associated with being a stakeholder surveyed on the CombinADO intervention package (R^2^ = 0.092, β = 1.56, 95% CI = 0.26–2.86, *p *= 0.02), being female (R^2^ = 0.057, β = 1.33, 95% CI = -0.06–2.71, *p* = 0.06), and having clinics with two or more HCWs delivering ART services to AYAHIV (R^2^ = 0.036, β = 1.40, 95% CI = -0.27–3.07, *p* = 0.10). No significant associations were observed between other considered predictors and the total attitude score. In the multivariable model, which included intervention type, gender, and number of HCWs at ART facilities for AYAHIV, we also included age as an explanatory of a priori interest. In this model, after controlling for other variables, only study package and the number of HCWs delivering ART care to AYAHIV remained significant to predict higher attitude scores among stakeholders (β = 1.57, 95% CI = 0.34–2.80, *p* = 0.01 and β = 1.57, 95% CI = 0.06–3.08, *p* = 0.04 respectively). R^2^ for this final model for predicting total attitude score with the four explanatory variables considered was higher than for each univariate model with significant predictors, at 0.2917 (*p* < 0.001).

**Table 4:** Linear regression: Association of individual and facility-level factors with overall attitude scores towards CombinADO intervention packages among stakeholders providing HIV care in AYAHIV in Nampula, Northern Mozambique, (*N* = 59)

Characteristic	Mean total score (SD); Range	Unadjusted linear regression			Adjusted multivariate linear regression****		
		**β Coefficient**	**95%CI**	***P*-value**	**β Coefficient**	**95%CI**	***P*-value**
Overall mean total attitude score	35.0 (2.59); 30–41						
***Stakeholder level***							
**Intervention group**							
ESOC package	34.24 (2.65); 30–41	**reference**	**reference**		**reference**	**reference**	
CombinADO package	35.8 (2.31); 31–41	**1.56**	**0.26**–**2.86**	0.02**	1.57	0.34–2.80	0.01***
**Age**		-0.07	-0.17–0.41	0.22			
24 and under	34.3 (3.21); 32–38	**reference**	**reference**		**reference**	**reference**	
25–34	35.3 (3.03); 31–41	0.97	-2.40–4.34	0.57	1.25	-2.63–5.13	0.52
35–44	35.3 (1.85); 32–39	0.97	-2.26–4.21	0.55	1.38	-2.45–5.21	0.47
45 and older	32 (1.83); 30–34	-2.33	-5.88–1.21	0.19	-1.99	-5.83–1.85	0.30
**Gender**							
Male	34.1 (2.40); 30–41	**reference**	**reference**		**reference**	**reference**	
Female	35.4 (2.59); 31–41	1.33	-0.06–2.71	0.06**	1.21	-0.35–2.78	0.13
**Current position**							
Medical doctor	34.8 (2.71); 30–40	**reference**	**reference**				
Nurse/midwife	34.9 (2.21); 31–38	0.07	-1.54–1.69	0.93			
Health tech	35.2 (3.14); 31–41	0.46	-1.54–2.46	0.65			
Other profession	35.7 (1.53); 34–37	0.83	-1.14–2.81	0.40			
**Primary role at the clinic**							
Health care worker (HCW)	35.1(2.44); 31–41	**reference**	**reference**				
Key informant (KI)	35 (2.72); 30–41	-0.08	-1.43–1.27	0.91			
Years working in the facility (SD), *n* = 26*		-0.10	-0.34–0.145	0.41			
Less than a year	35.8 (2.28); 33–38	**reference**	**reference**				
1 to 5	35.0 (2.77); 32–41	-0.80	-3.38–1.78	0.53			
6 or more	34.8 (2.19); 31–37	-1.05	-3.61–1.51	0.41			
***Facility level***							
**Location of facility**							
Rural	34.7(2.39); 30–39	**reference**	**reference**				
Urban	35.1(2.95); 31–41	0.45	-1.15–2.05	0.57			
Peri-urban	34.6(1.85); 31–37	-0.10	-1.80–1.61	0.91			
Governmental KI	36.3 (2.06); 34–39	1.52	-0.65–3.70	0.17			
**Facility size, *n* = 55**		0.79	-0.33–1.91	0.17			
Less than 200	34.7 (2.62); 30–41	**reference**	**reference**				
200 and more	35.3 (2.62); 31–41	0.61	-0.81–2.04	0.39			
**Number of HCWs at the facility**		0.00	-0.00–0.01	0.13			
1 HCW	33.8 (2.19); 31–36	**reference**	**reference**		**reference**	**reference**	
2 or more HCWs	35.1 (2.65); 30–41	1.40	-0.27–3.07	0.10**	1.57	0.06–3.08	0.04***

Null model: Explained facility-level variance without any variables (ICC,%) = Variance estimate /(Variance estimate + Explained variation) = (0.29/ 0.29 + 6.68) = 4.38%, *p* = 0.32

^*^Data only collected in HCWs

^**^p < 0.10

^***^p < 0.05

^****^For multivariable model: R^2^ = 0.2917, *p* < 0.001

## Discussion

This study aimed to examine the attitudes of HIV service providers towards adopting CombinADO interventions for improving HIV outcomes in AYAHIV receiving ART care in Mozambique. To our knowledge, this is the first study that considered the impact of service providers' attitudes toward adopting novel HIV interventions planned for services offered to AYAHIV in the SSA setting. Overall, service-providing stakeholders dedicated to HIV care of AYAHIV within this setting reported positive attitudes regarding adopting either the intervention or control CombinADO study intervention packages within health services. We found that the study package assessed (i.e., control or intervention condition) and the number of HCWs delivering ART care in participating clinics were the only significant explanatory variables to explain variations in stakeholder attitudes. Other individual and context-level factors most noted in the existing literature to influence providers' attitudes towards adoption in various interventions in different fields were not significant predictors in this setting.

Our study showed an overall positive attitudinal endorsement of adopting interventions tested for effectiveness in the CombinADO study. Since the stakeholders will ultimately be responsible for delivering them in actual practice, this is especially important. Previous studies have suggested that implementers tend to value needs-driven interventions since they incorporate feedback from priority populations and end-users on what would be most appropriate to address the identified contextual needs. Given that in developing the CombinADO intervention packages we aimed to address needs of AYAHIV, our results suggest that these stakeholders were already supportive of implementing these interventions within HIV services offered to AYAHIV in this setting, likely contributing to their positive attitudes [36, 38]. We recommend that as more researchers consider developing appropriate HIV interventions targeted at AYAHIV, they also consider incorporating the perspectives of service providers throughout the process. This may ensure that interventions are deemed by service providers, as ultimate implementers, to have value in intervening to meet the needs of AYAHIV in improving their HIV outcomes.

The results of the ARTAS questionnaire items showed that respondents broadly endorsed positive attitudes, as observed in the high mean values shared for each item used to measure attitudes. However, we noted that stakeholders in the CombinADO intervention package group were more likely than control package stakeholders to report that the intervention package would be easy to implement after training, which may explain the observed differences in total scores between intervention groups. Notably, in the CombinADO study, the intervention group package was developed to include six additional components to those in the ESOC package. Thus, it would be very reasonable for the stakeholders expected to implement this package to report higher needs for training to acquire the necessary skills and confidence to engage the different intervention components during implementation. Even with the high levels of attitudes reported in this study, it was essential to identify these differences in perspectives on the importance of training among stakeholders within different package groups prior to active implementation of the intervention in clinics because it assisted in scaling training based on the needs highlighted by stakeholders, thus supporting provider self-efficacy [19, 26]. Our study provides evidence of the usefulness of the ARTAS attitude subscale in understanding stakeholders' attitudes towards intervention adoption. In addition, we recognize its benefit in identifying factors relating to intervention content and structure that may hinder the successes of implementation efforts beyond adoption that may require attention before the active implementation of interventions in health services.

Our results also showed an association between the number of HCWs delivering ART care in clinics and stakeholder attitude scores. It was less surprising to observe that having one HCW at the clinic offering services to AYAHIV would predict lower attitudinal scores towards adopting new interventions in practice, especially given the multi-component nature of packages tested in the CombinADO study. It has previously been suggested that service providers may be reluctant to implement new interventions if they add to their workload, especially when inadequate personnel are available. Existing implementation literature has highlighted that adequate resources, such as training and staffing, are necessary to successfully adopt new EBI in healthcare settings [46, 47]. The mechanisms by which they influence providers' attitudes toward adopting EBI may be complex and beyond the scope of this article; however, studies have noted that these factors can impact readiness to implement new EBIs [32, 46, 47]. As a result, adequacy of resources may partly explain the significant variation in stakeholder attitudes in this study due to the number of health care workers in practice and training requirements related to the type of package proposed. We recommend that before implementing new interventions in healthcare, especially in resource-limited settings, researchers should consider the role of resources such as training and staffing when addressing barriers.

In contrast to other studies on the subject, we found no additional significant relationship between reported provider attitudes and the remaining individual and facility-level characteristics considered in the study [26, 27, 31, 48]. First, there may be more complex factors influencing providers' attitudes in this setting that were not investigated in this study, and second, this could be explained in part by a lack of adequate power to show any associations that may have existed between different stakeholder groups due to the small sample size of our study. One interesting pattern that emerged was a change in the direction of the relationship between stakeholder attitudes and the age variable. Though not statistically significant in our study, we found that older stakeholders had less favourable attitudes toward intervention adoption. Previous research has revealed a similar relationship pattern and provided several explanations [31, 48, 49]. According to studies, younger stakeholders are generally in the early stages of their careers and may be more open to learning new skills and acquiring new knowledge than their older counterparts who may value their "traditional" practices [26, 49]. Because our study sample was generally younger, it is possible that our study was underpowered to demonstrate the significance of these age differences. A similar pattern was observed for the clinic location variable. Stakeholders in peri-urban clinics reported less favourable attitudes toward intervention adoption when compared to stakeholders representing clinics in other geographic locations. Although this is beyond the scope of this study, we recognize that this relationship may merit further investigation in future studies. Nonetheless, we believe these findings highlight the importance of understanding how various individual and facility-level characteristics may influence intervention adoption in each setting.

The major strength of this study lies in the purposive sampling and the use of a validated measure to understand better key stakeholders' attitudes who may influence whether these interventions reach AYAHIV in the future if interventions are found effective in improving their HIV outcomes. Given our study results, we could streamline training needs and consider additional human resource support needed to promote adoption in clinics before moving forward with active implementation. However, we acknowledge several limitations of our study. First, the self-report nature of our study is prone to social desirability bias, a problem commonly found in self-report studies, which may have led to higher attitudes towards adopting interventions reported by providers than they felt. Second, given our study was cross-sectional, we could not ascertain the temporality of provider attitude in the long term; however, we plan to re-examine these in the later stages of the study. Third, we acknowledge that using quantitative methods and including a priori explanatory variables may not have allowed us to capture additional perspectives on factors that may have been critical in this setting to shape individual providers' attitudes toward adopting these interventions. We recommend that future studies consider using various research methods, such as mixed methods, to better understand the complexities of individual attitudes. Finally, given the sample size, our data may not be generalizable to other settings; thus, future studies should consider assessing provider attitudes based on their unique interventions and contextual factors. However, we believe that the data shared in this study are the beginning of understanding approaches that can be used and considered in future implementation studies within similar settings to assess the role of key stakeholders' attitudes in the success of intervention implementation efforts. We also believe this study helped with the future direction of our research. We think it would be beneficial to understand how providers' attitudes would have changed in the active implementation of the intervention packages in clinics. In addition, we will consider using various research methods, such as mixed methods, to better understand the complexities related to the attitudes of individual providers in this setting. Such efforts may help identify barriers that in the future may hinder moving towards successful sustainability and scale-up of these interventions within this setting.

## Conclusion

This study found positive attitudes toward adopting CombinADO study interventions among HIV care providers for AYAHIV in Nampula, Mozambique. According to this study, developing needs-based interventions and understanding the contextual factors before active implementation is key to promoting HIV interventions in healthcare settings. Including service providers in developing interventions addressing their clients' needs and considering factors specific to their work context is likely to improve attitudes towards interventions and, accordingly, the adoption of interventions. Furthermore, our study shows that perceived adequacy of resources―in this study, training and human resources―may be necessary for shaping attitudes towards adopting new interventions in this and similar healthcare settings. Achieving success in the implementation process of novel interventions, especially those with complex components, requires understanding implementers' perspectives, as well as their characteristics and their work contexts.

## Data Availability

Data collection tools are available upon reasonable request to the corresponding author or contacts listed in Clinicaltrials.gov.

## Abbreviations

ART: Antiretroviral therapy
AYAHIV: Adolescents and young people living with HIV
AYFS: Adolescent and youth-friendly services
cRCT: Cluster-randomized trial
EBI: Evidence-based interventions
ESOC: Enhanced standard of care
HCW: Healthcare worker
IRB: Institutional Review Board
KI: Key informant
MISAU: Mozambique Ministry of Health
NICHD: National Institute of Child Health & Human Development
PATC3H: Prevention and Treatment through a Comprehensive Care Continuum for HIV-affected Adolescents in Resource-Constrained Settings
SSA: sub-Saharan Africa
